# Differences in the Clinical Outcome of Osteomyelitis by Treating Specialty: Orthopedics or Infectology

**DOI:** 10.1371/journal.pone.0144736

**Published:** 2015-12-17

**Authors:** Carolina Arias Arias, Maria Carolina Tamayo Betancur, Miguel Alejandro Pinzón, Doris Cardona Arango, Cesar Antonio Capataz Taffur, Edgar Correa Prada

**Affiliations:** 1 Department of Epidemiology, Universidad CES, Medellín, Antioquia, Colombia; 2 Department of Infectious Diseases, Clínica Medellín, Medellín, Antioquia, Colombia; 3 Department of Infectious Diseases, Fundación Clínica del Norte, Bello, Antioquia, Colombia; 4 Department of Orthopedics, Nueva Clínica Sagrado Corazón, Medellín, Antioquia, Colombia; University of Oulu, FINLAND

## Abstract

Osteomyelitis is a heterogeneous infection with regard to etiology and treatment, and currently no single management protocol exists. Management of the condition is typically an interdisciplinary approach between orthopedics and infectious disease; however, the orthopedist is often the person who manages treatment. The aim of the study was to determine differences in the outcome of osteomyelitis according to its treating specialty and to identify factors associated with the recurrence of the disease. An ambispective cohort study of 129 patients with osteomyelitis was conducted and the proportions for qualitative variables and central tendency and dispersion measures for quantitative variables were calculated; the latter were tested for normality using the Shapiro-Wilk test. A bivariate analysis was conducted with measures of association based on the chi square test and crude relative risk. A logistic regression model was applied and statistical significance was set at p < 0.05, including the model of relevant clinical variables that fit the Hosmer-Lemeshow test. We found that 70% of patients were treated either by orthopedics or infectious disease. Patients who were treated by an orthopedist alone presented a greater risk of relapse or reinfection (RR = 4.6; 95% CI 2.3;8.9). Risk factors of osteomyelitis recurrence as determined in the regression model included the following: age of 57 years or older (RR = 1.3; 95% 0.3;5.2), long bones (RR = 1.9; 95% CI 0.5;7.1), fracture (RR = 5.0; 95% CI 0.4;51.4), monotherapy (RR = 3.0; 95% CI 0.6;14.5), receiving less than 4 weeks of antibiotics (RR = 1.5; 95% CI 0.2;10.1), inadequate treatment (RR = 3.1; 95% CI 0.4;20.1), and receiving orthopedics treatment (RR = 5.5; 95% CI 1.6;18.2). Most patients evaluated jointly by orthopedics and infectious disease received adequate treatment for osteomyelitis and had fewer relapses.

## Introduction

Osteomyelitis is an inflammatory process that affects bone due to the contiguous infection, direct inoculation, or hematogenous spread of microorganisms [1]. Current interest in this condition has increased due to recent changes in the epidemiology, pathogenesis, diagnosis, treatment, and prognosis of the disease [2, 3]. The reported incidence has increased due to comorbidities such as diabetes mellitus, peripheral vascular disease, trauma and surgery [4]. After an open fracture, the incidence of osteomyelitis can range from 2% to 16% depending on the type of injury and the treatment administered [5].

Despite the significant progress made in recent decades for its management, the optimal medical-surgical treatment of choice remains largely unknown [6]. Current literature is not sufficient to determine the best antimicrobial agent to use, route of administration, or duration of treatment [7]. Management should be multidisciplinary between orthopedics and infectious disease with the aim of combining surgical techniques with the appropriate antimicrobial agent that favors clinical success [8]; however, in some institutions, it is common for the orthopedist to be responsible for treatment, including the prescription of antimicrobial agents, which could lead to inappropriate and indiscriminate use of antibiotics due to unawareness of the microorganism [9]. This inappropriate approach can often lead to microbial resistance, treatment failure, and drug toxicity.

Although the treatment of other infectious entities by infectious disease specialists has been associated with improved clinical outcomes, information regarding osteomyelitis remains limited [10]. However, the creation of clinical protocols that standardize the joint management by both specialties could improve the treatment of osteomyelitis. This would also identify the best medical-surgical treatment of choice, the optimal dose and type of antimicrobial to be used, and the most appropriate route of administration and duration of therapy, thus benefiting both the patient and the entire healthcare system.

To the best of our knowledge, no studies to date have compared the outcome of osteomyelitis treatment based on the treating specialty. Therefore, the aim of this study was to determine the differences in the outcome of treating osteomyelitis according to its treating specialty and identify factors associated with the recurrence of the disease.

## Methods

This study was an ambispective cohort study of 129 clinical records obtained by census in two hospitals in the city of Medellín, Colombia. Patients were 18 years of age and older who had been diagnosed with osteomyelitis by bone culture between 2013 and 2014. The clinical history of patients was revised and followed-up after three, six, and twelve months after hospital discharge to determine the outcome of the infection; The follow-up ended when relapse or reinfection was documented. The study included patients who were diagnosed with osteomyelitis of the short, long, flat, and sesamoid bones (except face and vertebrae), with or without osteosynthesis material and/or prosthesis, of any microbial etiology, diagnosed by bone culture, and treated by orthopedics only or jointly with infectious disease. Only patients whose medical and microbial records were readily available for data collection were included in this study. We excluded patients with a life expectancy of six months or less, those who did not start treatment, and those who had records with insufficient information.

The dependent variables were as follows: *a)* Cure: absence of evidence of bone disease (signs and symptoms of osteomyelitis) such as pain, fistula, secretion, edema, erythema, or local heat; *b)* Relapse: reappearance of signs and symptoms of disease plus one or more positive bone cultures for the previously isolated and treated microorganism; and *c)* Reinfection: reappearance of the signs and symptoms of the disease plus one or more positive cultures for different microorganisms from the initially isolated and treated one.

The following variables were assessed: demographics (age, gender, and affiliation to the social security health system), clinical (personal history of disease, microorganism, treatment received, days of treatment, bone exposure, length of hospital stay, treating specialty and proper treatment is defined as: use of antimicrobial for which the organism was susceptible according to antibiogram, antibiotic treatment for 4 weeks or longer, or shorter in cases of amputation above the Affected bone level.), and surgical (number of required surgeries, presence and/or removal of the osteosynthesis material and/or prosthesis, and need for amputation).

Data were processed using SPSS software version 21.0 (SPSS Inc, Chicago, Illinois, USA) and STATA version 12.0 (STATA Inc, College Station, Texas, USA). Licenses were covered by CES University. In the univariate analysis, proportions for qualitative variables and measures of central tendency were calculated, and dispersion and position for quantitative variables were also assessed; a Shapiro-Wilks normality test was applied to the latter. For the bivariate analysis, some variables were recoded based on the literature and clinical experience in order to facilitate their analysis and address any possible confusion. The chi-square test was used to determine associations and crude Relative Risk (RR) Relative with respective confidence intervals at 95% were calculated. Statistical significance was set at p < 0.05.

Other variables with underreporting > 10% were eliminated, such as: "nutritional status" and "collection and culture method". Therefore, they were not taken into account in the analysis.

The variables that reached statistical significance and those that were relevant to the study and fit the Hosmer-Lemeshow criteria entered the logistic regression model, from which the adjusted RR was obtained for each of these variables and the behavior related to the clinical outcome of osteomyelitis was determined. The project was approved by the Institutional Research Ethics Committee in humans of CES University (session number 71, code project 314) and the Operational Research Committee of CES University. This is a study without risk because the information was from secondary sources from clinical records and no consent was given by this reason, the data were analized anonymously.

## Results

**Fig 1:**
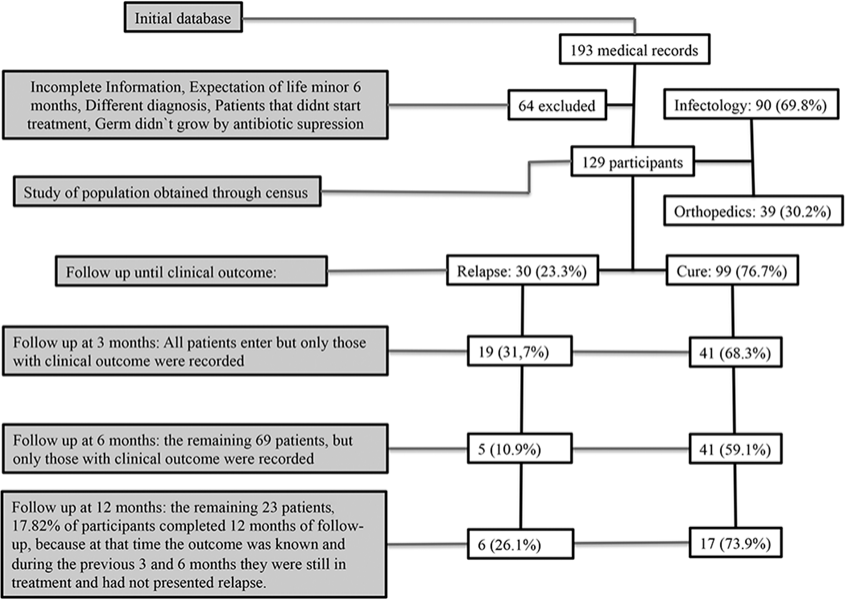
Flowchart of patients and follow up. The patients that presented relapse didn´t continue follow up because they weren´t part of the interest of the study.

From the initial database consisting of 193 medical records of patients diagnosed with osteomyelitis, 129 medical records met inclusion criteria for the study. Sixty-four records were excluded for one or more of the following reasons: no evidence of culture, cultures without microbial growth, diagnoses other than osteomyelitis, or osteomyelitis of the face or vertebra. In addition, clinical histories were discarded if there was a lack of follow-up monitoring (Fig 1). Of the 129 patients on study, the age ranged from 18 to 91 years and 104 (80.6%) were male. In addition, 50% of patients were 42 years of age or younger and 102 (79.1%) had no significant medical history. Of those who did report some background, 14 (51.8%) had two or more diseases (metabolic and vascular) and 7 (25.9%) had diabetes mellitus type 2.

Sixty-two percent of patients had osteosynthesis material (OSM) at the time of infection diagnosis and 62 (77.5%) had theirs removed as part of the treatment. The remaining patients maintained the material with antimicrobial salvage therapy. The predominant cause of osteomyelitis was bone fracture 115 (89.2% of cases). (See S1 Database for additional information).

The most common infection was polymicrobial infection 40 (31.0%) followed by methicillin-susceptible Staphylococcus aureus (MSSA) 37 (28.7%; Table 1).

**Table 1:** Percentage distribution of patients with osteomyelitis according to the isolated organism in culture.

Isolated germ in culture	No	%
*Polymicrobial*	40	31.0
*Methicillin-susceptible Staphylococcus aureus (MSSA)*	37	28.7
*Methicillin-resistant Staphylococcus aureus (MRSA)*	12	9.3
*Non extended-spectrum beta-lactamase-producing enterobacteriaceae (No ESBL)*	8	6.2
*Pseudomonas aeruginosa*	7	5.4
*AmpC beta-lactamases enterobacteriaceae*	5	3.9
*Other coagulase-negative Gram-positive Cocci*	5	3.9
*Enterococcus faecalis*	4	3.1
*Methicillin-resistant Staphylococcus epidermidis (MRSE)*	3	2.3
*Methicillin-susceptible Staphylococcus epidermidis (MSSE)*	3	2.3
*Extended-spectrum beta-lactamase-producing enterobacteriaceae (ESBLs)*	2	1.6
*Klebsiella pneumoniae carbapenemase type enterobacteriaceae* * *(KPC)*	1	0.8
**TOTAL**	129	100.0

* Confirmed by a positive modified Hodge test

Joint therapy of two or more antimicrobial strategies was the most frequently used treatment 113 (87.6% of cases). In addition, 103 (79.8%) of patients received appropriate antimicrobial therapy at the discretion of an outside expert who did not know the identification of the treating specialty. Of the patients who received antimicrobial therapy from an infectious disease specialist, 75 (83.3%) completed 4–6 weeks of therapy, while 23 (59.0%) of patients treated by an orthopedist received less than 4 weeks of antibiotics. Approximately 90 (70%) of patients were treated concomitantly between the orthopedic and infectious disease departments (Table 2).

**Table 2:** Percentage distribution of patients with osteomyelitis based on demographics and clinical characteristics, grouped by treating specialty.

Demographic and clinical data		Infectology* (n = 90)		Orthopedics (n = 39)		Total (n = 129)
		No	%	No	%	No
**Sex**	Male	69	76.7	35	89.7	104
**Affiliation**	Contributory	58	64.4	17	43.6	75
**Osteosynthesis material**	Yes	59	65.6	21	53.8	80
**Removal oh osteosynthesis material** **	Yes	47	79.6	15	71.4	62
**Treatment**	Combined therapy	82	91.1	31	79.5	113
**Appropriate treatment**	Yes	85	94.4	18	46.2	103
**Exposed bone**	Yes	33	36.7	10	25.6	40
**Covered defect** **	Yes	31	93.9	9	90.0	39
**Hospital stay**	More than 14 days	66	73.3	25	64.1	91
**Clinical outcome**	Cure	80	88.9	19	48.7	99
	Relapse	6	6.7	17	43.6	23
	Reinfection	4	4.4	3	7.7	7

* The group infectology that Refers to those patients received initial treatment by a professional orthopedic, their antimicrobial treatment but was only addressed by the infectious disease specialist (multidisciplinary approach).

** Proportional distribution of patients that had osteosynthesis material or exposed bone.

OSM was present at the time of diagnosis in 59 (65.6%) of patients treated by infectious disease and 21 (53.8%) of patients treated by orthopedics. Moreover, 47 (79.6%) of the patients treated by infectious disease and 15 (71.4%) treated with orthopedics had their OSM removed as part of treatment. In addition, 82 (91.1%) of patients treated by an infectious disease specialist received combined therapy (two or more antibiotics) for the treatment of osteomyelitis, while 8 (20.5%) of patients treated by an orthopedist received monotherapy. In total, 85 (94.4%) of patients treated by infectious disease patients received appropriate antimicrobial therapy, while only 18 (46.2%) of patients treated by orthopedics received the appropriate therapy.

We found that 80 (88.9%) of patients evaluated by infectious disease were cured, while 17 (43.6%) of patients treated by orthopedics relapsed. Moreover, 88 (88.9%) of those who achieved a cure received adequate treatment. For patients who were cured of osteomyelitis, 78 (78.8%) completed 4–6 weeks of antibiotic therapy (Table 3).

**Table 3:** Percentage distribution of patients with osteomyelitis based on clinical outcome: cure and recurrence by demographic and clinical data.

Demographic and clinical data		Outcome				Total (n = 129)
		Cure (n = 99)		Recurrence (n = 30)		
		No	%	No	%	
**Sex**	Male	78	78.8	26	86.7	104
**Triggering cause**	Hematogenous	5	5.1	1	3.3	6
	Open fracture	38	38.4	20	66.7	58
	Closed fracture	48	48.5	9	30.0	57
	Diabetic foot	3	3.0	0	0.0	3
	Chronic occlusive arterial disease (COAD)	3	3.0	0	0.0	3
	Infected contiguous focus extension	2	2.0	0	0.0	2
**Medical history**	No history of importance	79	79.8	22	73.3	101
**Osteosynthesis material**	Yes	59	59.6	21	70.0	80
**Removal of osteosynthesis material**	Yes	47	79.6	15	71.4	62
**Treatment**	Combined therapy	91	91.9	22	73.3	113
**Appropriate treatment**	Yes	88	88.9	15	50.0	103
**Duration of treatment**	Less than 4 weeks	11	11.1	8	26.7	19
	4 to 6 weeks	78	78.8	20	66.7	98
	More than 6 weeks	10	10.1	2	6.7	12
**Group of effected bones**	Long	67	67.7	24	80.0	91
	Short	13	13.1	1	3.3	14
	Flat	7	7.1	3	10.0	10
	Sesamoid	12	12.1	2	6.7	14
**Exposed bone**	Yes	34	34.3	9	30.0	43
**Covered defect**	Yes	32	94.1	8	88.8	40
**Treating specialty**	Infectology	80	80.8	10	33.3	90

We also found that 80 (80.8%) of patients that achieved a cure received antibiotic treatment from an infectious disease specialist. Of the patients who had recurrence, 10 (33.3%) had an MSSA infection and 7 (23.3%) had a polymicrobial infection (Table 4).

**Table 4:** Percentage distribution of patients with osteomyelitis based on clinical outcome: cure and recurrence grouped by microorganism identified.

Isolated microorganism	Cure (n = 99)		Recurrence (n = 30)		Total (n = 129)
	No	%	No	%	No
*Methicillin-susceptible staphylococcus aureus (MSSA)*	27	27.3	10	33.3	37
*Repressed AmpC beta-lactamases enterobacteriaceae*	2	2.0	3	10.0	5
*Unrepressed AmpC beta-lactamases enterobacteriaceae*	0	0.0	2	6.7	2
*Klebsiella pneumoniae carbapenemase type enterobacteriaceae* * *(KPC)*	1	1.0	0	0.0	1
*Polymicrobial*	33	33.3	7	23.3	40
*Methicillin-resistant Staphylococcus aureus (MRSA)*	10	10.1	2	6.7	12
*Methicillin-resistant Staphylococcus epidermidis (MRSE)*	1	1.0	2	6.7	3
*Methicillin-susceptible Staphylococcus epidermidis (MSSE)*	3	3.0	0	0.0	3
*Enterococcus faecalis*	3	3.0	1	3.3	4
*Other coagulase-positive Gram-positive Cocci*	4	4.0	1	3.3	5
*Pseudomonas aeruginosa*	6	6.1	1	3.3	7
*Non extended-spectrum beta-lactamase-producing enterobacteriaceae (No ESBL)*	7	7.1	1	3.3	8
*Extended-spectrum beta-lactamase-producing enterobacteriaceae (ESBLs)*	2	2.0	0	0.0	2

* Confirmed by a positive modified Hodge test.

Regarding the outcome of the disease, which was the main focus of the study, 99 (76.7%) of patients were cured while 23 (17.8%) relapsed and 7 (5.4%) experienced reinfection. A total of 23 (17.82%) of the patients received follow-up at 12 months post-treatment and 17 (73.91%) of these patients achieved a cure (Table 5).

**Table 5:** Percentage distribution of patients with osteomyelitis based on clinical outcome: cure, relapse, and reinfection grouped by follow-up time.

Follow-up time	Clinical outcome						Total
	Cure		Relapse		Reinfection		
	No	%	No	%	No	%	
**3 months**	41	41.4	17	73.9	2	28.6	60
**6 months**	41	41.4	3	13.0	2	28.6	46
**12 months**	17	17.2	3	13.0	3	42.9	23
**Total**	99	100.0	23	100.0	7	100.0	129

Of 39 patients treated by orthopedics, 21 (53.8%) received inadequate antibiotic treatment. This finding was determined by an expert who took into account not only the antimicrobial activity of the isolated microorganism, but also the duration of the antibiotic treatment in cases when the affected bone was preserved and when an amputation was necessary. Infectious disease prescribed antibiotics to 90 patients and the independent expert determined that 85 (94.4%) of these patients received adequate treatment for the management of infection.

### Factors associated with recurrence of osteomyelitis

The type of isolated microorganism did not have a significant association with outcome. We found that 8 (26.7%) of those who had recurrence received monotherapy, which, although not a significant variable, was a factor that increased the likelihood of osteomyelitis recurrence. Regarding the duration of treatment, 8 (26.7%) of those who relapsed received less than four weeks of antimicrobial therapy and 2 (6.7%) completed more than six weeks. This relationship was not statistically significant. Moreover, 15 (50%) of patients that had recurrence did not receive adequate treatment for the infection (p = 0.000); patients who did not receive adequate treatment have 3.9 times the risk of recurrence than those who did receive proper treatment (Table 6).

**Table 6:** Association between social and clinical characteristics of patients diagnosed with osteomyelitis and clinical outcome after treatment of the infection.

Social and clinical variables		Clinical outcome				X^2^ test	P value	RR crude	95% CI
		Recurrence		Cure					
		No	%	No	%				
**Age**	Under 29 years	9	30.0	23	23.2	4.509	0.211	1	-
	30 to 41 years	3	10.0	28	28.3			0.274	0.066, 1.131
	42 to 56 years	7	23.3	22	22.2			0.813	0.258, 2.562
	57 years or over	11	36.7	26	26.3			1.081	0.380, 3.073
**Sex**	Male	26	86.7	78	78.8	0.915	0.339	1.563	0.600, 4.072
	Female	4	13.4	21	21.2			1	-
**Affiliation**	Subsidized	12	40.0	42	42.4	0.056	0.814	0.926	0.488, 1.758
	Contributory	18	60.0	57	57.6			1	-
**Background (diseases)**	Metabolic and vascular	4	13.4	4	4.0	**Fisher	0.840	2.327	1.075, 5.036
	Other background	26	86.7	95	96.0			1	-
**Cause of osteomyelitis**	Fractures	29	96.7	86	86.9	**Fisher	0.186	3.530	0.520, 23.954
	Other causes	1	3.3	13	13.1			1	-
**Affected bone**	Long bones	24	80.0	67	67.7	1.683	0.195	1.670	0.743, 3.757
	Others bones	6	20.0	32	32.3			1	-
**Presence of osteosynthesis material**	Yes	21	70.0	59	59.6	1.058	0.304	1.429	0.713, 2.864
	No	9	30.0	40	40.4			1	-
**Removal of osteosynthesis material**	No	5	23.8	12	20.3	**Fisher	0.479	1.158	0.495, 2.708
	Yes	16	76.2	47	79.7			1	-
**Isolated germ in culture**	Gram-positive Cocci	16	53.3	48	48.5	0.217	0.642	1.161	0.619, 2.177
	Enterobacteriaceae	14	46.7	51	51.5			1	-
**Type of treatment**	Monotherapy	8	26.7	8	8.1	**Fisher	0.120	2.568	1.386, 4.760
	Combined therapy	22	73.3	91	91.9			1	-
**Weeks of treatment**	Less than 4 weeks	8	26.7	11	11.1	4.071	0.131	0.275	0.047, 1.615
	4 to 6 weeks	20	66.7	78	78.8			0.780	0.158, 3.847
	6 weeks or more	2	6.7	10	10.1			1	-
**Appropriate treatment**	No	15	50.0	11	11.1	21.636	0.000*	3.962	2.236, 7.019
	Yes	15	50.0	88	88.9			1	-
**Treating specialty**	Orthopedics	20	66.7	19	19.2	24.602	0.000*	4.615	2.386, 8.926
	Infectology	10	33.3	80	80.8			1	-
**Follow-up time**	3 months	19	63.3	41	41.4	6.976	0.031*	0.762	0.259, 2.238
	6 months	5	16.7	41	41.4			2.894	0.777, 10.776
	12 months	6	20.0	17	17.2			1	-
**Hospital stay**	Up to 14 days	10	33.3	28	28.3	0.283	0.595	1.197	0.620, 2.312
	More than 14 days	20	66.7	71	71.7			1	-

* Statistically significant association with p < 0.05

** Fisher Association Test

The treating specialty was found to be a risk factor for recurrence of the disease: patients who were treated only by orthopedics had a 4.6 times greater risk of recurrence than those who were treated concomitantly by orthopedics and infectious disease.

Although only three variables in the study reached statistical significance, those that had a p value less than 25% as assessed by the Hosmer-Lemeshow test as well as those of clinical importance according to the literature and clinical experience were included in the model.

All variables, including age, affected bone, osteomyelitis cause, type of treatment, duration of treatment, appropriate treatment, and treating medical specialty were found to be risk factors for disease recurrence. Although the 95% CI did not reach statistical significance for any variable, the findings were considered significant when analyzed based on clinical experience, and this is explained by the low number of patients treated by orthopedic. With respect to age, we found that people who were 57 years of age or older had 1.3 times the risk of relapse compared to patients who were 29 years old or younger (95% CI 0.339–5.292). The crude RR did not change when adjusted (Table 7).

**Table 7:** Calculation of crude RR crude adjusted for variables associated with the clinical outcome of osteomyelitis.

Social and clinical variables		RR crude	95% CI	RR adjusted	95% CI
			Lower—Upper		Lower—Upper
**Age**	Under 29 years	1	-	1	-
	30 to 41 years	0.274	0.066, 1.131	0.265	0.052, 1.347
	42 to 56 years	0.813	0.258, 2.562	0.886	0.210, 3.743
	57 years or over	1.081	0.380, 3.073	1.340	0.339, 5.292
**Affected bone**	Long bones	1.670	0.743, 3.757	1.944	0.527, 7.170
	Other bones	1	-	1	-
**Cause of osteomyelitis**	Fracture	3.530	0.520, 23.954	5.021	0.494, 51.062
	Other causes	1	-	1	-
**Type of treatment**	Monotherapy	2.568	1.386, 4.760	3.070	0.650, 14.506
	Combined therapy	1	-	1	-
**Weeks of treatment**	Less than 4 weeks	0.275	0.047, 1.615	1.569	0.243, 10.134
	4 to 6 weeks	0.780	0.158, 3.847	0.845	0.050, 14.232
	More than 6 weeks	1	-	1	-
**Appropriate treatment**	No	3.962	2.236, 7.019	3.173	0.499, 20.180
	Yes	1	-	1	-
**Treating Specialty**	Orthopedics	4.615	2.386, 8.926	5.552	1.688, 18.267
	Infectology	1	-	1	-

Patients who had involvement of the long bones had 1.9 times the risk of relapse or reinfection than those with other groups of bones affected. Moreover, if the cause of osteomyelitis was a fracture, then these patients had 5 times the risk of recurrence of infection. Patients treated with monotherapy had 3 times the risk of recurrence compared to those receiving combined therapy. Patients treated with antibiotics for less than four weeks had 1.5 times the risk of recurrence compared to those treated for more than six weeks. The crude RR in this case was a factor that protected recurrence, but once the variables were set, it behaved as a risk factor (95% CI 0.243–10,134). In addition, patients who received inadequate treatment had 3.1 times the risk of relapse. Patients treated only by orthopedics had a 5.5 times higher risk of recurrence of osteomyelitis.

In the final logistic regression model, the treating medical specialty variable was the only one that reached statistical significance and was identified as a risk factor. Patients treated by orthopedics alone had a greater risk of disease recurrence. However, the findings of other variables may be affected by the sample size, which was one of the limitations of our study and a point that could be evaluated in future studies (Table 8).

**Table 8:** Final regression model. Clinical outcome of osteomyelitis and factors associated with recurrence.

Variable	P value	RR	CI 95% Exp (β)
			Lower—Upper
**Age (Up to 29 years)**	0.304	-	-
**Age (30–41 years)**	0.109	0.265	0.052, 1.347
**Age (42–56 years)**	0.869	0.886	0.210, 3.743
**Age (57 years and over)**	0.677	1.340	0.339, 5.292
**Affected bone (long bones)**	0.318	1.944	0.527, 7.170
**Cause of osteomyelitis (Fracture)**	0.173	5.021	0.494, 51.062
**Type of treatment (monotherapy)**	0.157	3.070	0.650, 14.506
**Weeks of treatment (more than 6 weeks)**	0.773	-	-
**Weeks of treatment (less than 4 weeks)**	0.636	1.569	0.243, 10.143
**Weeks of treatment (4 to 6 weeks)**	0.907	0.845	0.050, 14.232
**Appropriate treatment (No)**	0.221	3.173	0.499, 20.180
**Treating specialty (orthopedics)**	0.005	5.552	1.688, 18.267

## Discussion

Osteomyelitis is a heterogeneous disease regarding its etiology and treatment, and therefore it is difficult to perform well-designed, randomized, and controlled studies that compare outcome according to the antimicrobial therapy [11] used and factors associated with relapse. It is also challenging to determine whether the care a patient receives regarding the infectious disease has any impact on its outcome. In the present study, intervention by an infectious disease specialist was associated with a higher cure rate and lower risk of relapse, whereby the risk of osteomyelitis recurrence (relapse or reinfection) was 5.5 times greater in patients receiving antimicrobial therapy ordered by an orthopedist.

This study found that polymicrobial infection was the most common 40 (31.0%) in patients with osteomyelitis followed by MSSA 37 (28.7%), which is a finding similar to that reported in the literature where *S*. *aureus* and *P*. *aeruginosa* are the most frequently observed [12].

We found that 30 (23.2%) of patients relapsed, which is lower than the historical rate of relapse or clinical failure reported by Waldvogel (30–40%) [13]. This difference may be explained by the type of treatment received, safety of the therapy, and/or the osteosynthesis material present. In a retrospective cohort of 124 patients with OM and septic arthritis due to methicillin-sensitive *S*. *aureus*, Wieland [14] reported clinical failure in 23% of patients (after a follow-up period of six months) treated with ceftriaxone and a failure of 19% in patients treated with oxacillin, which are similar rates to those found in this study. However, in that cohort all patients were evaluated by the infectious disease department and the authors only compared clinical outcome based on the antimicrobial treatment prescribed.

In a study by Salvana et al [15], 82 patients with osteomyelitis were treated by a multidisciplinary medical team consisting of an orthopedic surgeon, a specialist in infectious disease, a plastic surgeon, and a nurse over a period of seven years. The average number of surgeries per patient was 2.2 with administration of intravenous antibiotics for two weeks and 60 days of oral antibiotics as directed by infectious disease, and only one case experienced recurrence in the first six months. This finding is on the lowest rates of treatment failure reported to date. However, cases treated exclusively by orthopedics were not evaluated.

Prior to this study, the clinical impact of intervention by an infectious disease specialist in osteoarticular pathologies had not been compared directly with management provided by orthopedics. The study by Uçkay et al [16] was the only study that showed that daily evaluation by an infectious disease specialist in an orthopedics unit coincided with reduced consumption of antibiotics, adjust therapy in a targeted manner with reduced costs, and no change in the rate of recurrence. However, control over the antibiotics prescription and differences in management requirements among different specialties were not assessed directly.

Among the findings of our study, the better clinical cure rate and lower recurrence rate in patients treated by the infectious disease department can be explained in part by the inappropriate use of antibiotics by orthopedics (inadequate treatment was associated with a 3.1 times greater risk of disease recurrence), greater percentage of use of combined antibiotic therapy by infectious disease specialists (patients receiving a monotherapy had 3.0 times the risk of recurrence), and longer duration of antimicrobial therapy prescribed by that specialty (patients who received less than four weeks of antibiotics had a 1.5 times greater risk of disease recurrence compared to those who received more than six weeks of antibiotics). Although these factors were not statistically significant in the multivariate analysis, they did become relevant according to clinical experience.

In 1986, Norden conducted one of the first randomized, controlled, and multicenter studies on the treatment of osteomyelitis. He recruited 18 patients between 1980 and 1982, of which eight received nafcillin and ten received nafcillin plus rifampin for 42 days. Only 50% of patients receiving monotherapy with nafcillin achieved a cure, while eight of ten patients (80%) in the combined therapy group achieved clinical success. However, due to the small number of patients in the study, differences between monotherapy and combined antibiotic therapy for osteomyelitis could not be demonstrated [17].

Sheftel and Mader assessed the clinical response of 18 patients with osteomyelitis due to enterobacteriaceae, including *P*. *aeruginosa*. They divided the patients into two groups: the first group received ceftazidime for 42 days and the second group received combined therapy with tobramycin and ticarcillin for 42 days. Of the nine patients receiving ceftazidime, three (33%) presented clinical failure or no improvement. In contrast, the nine patients (100%) receiving combined therapy with tobramycin and ticarcillin showed clinical improvement [18]. Van Der Auwera et al also conducted a double-blind, placebo-controlled study of oxacillin or vancomycin alone or in combination with rifampicin for the clinical outcome of various infections by *S*. *aureus*, including infections such as bacteremia, pneumonia, osteomyelitis, and wound infections. Of 65 patients, 23 had osteomyelitis: 10 patients received combined therapy of two antibiotics and 13 received monotherapy. Only one clinical failure occurred in the combined group compared to five failures in the monotherapy group. The average treatment time was 21 days and the clinical response rate was determined at hospital discharge without follow-up [19]. Although the trend in these studies indicates a better outcome using combined therapy, the small number of patients enrolled in each study, different times of treatment and follow-up, and different definitions for cure and relapse make it difficult to determine whether there is a real benefit of combined therapy for the management of osteomyelitis.

Regarding the duration of treatment, a retrospective study of 253 patients with vertebral osteomyelitis found that the duration of an antimicrobial treatment of 4 weeks or less of intravenous therapy was associated with a higher relapse rate [20]. In this study we did not include patients with vertebral osteomyelitis.

No association between microbial etiology and risk of relapse was observed regardless of the type of antibiotic used, which is a similar finding as reported by Tice et al [21]. However, the authors of the previous study found an increased risk of recurrence in cases of bone infection by *P*. *aeruginosa* (RR = 2.5, 95% CI 1.3–4.7 p = 0.005) and patients treated with vancomycin (RR = 3) for *MRSA*.

Tice and colleagues evaluated 254 patients with osteomyelitis and found that most relapses (95%) occurred in the first 6 months after the first cycle of antibiotic therapy. Our findings are consistent with Tice, since 80% of those who had disease recurrence experienced it in the first 6 months of follow up. Mader et al. [22] proposed a minimum follow-up period of one year to define cure or relapse of osteomyelitis in order to accelerate the development of new molecules for the treatment of this pathology. Nevertheless, as we and others have shown, it appears that the follow-up time could be reduced to achieve that objective.

The results of our study suggest that joint management of osteomyelitis patients by infectious disease specialists and orthopedists improves the clinical outcomes. In the absence of further studies to confirm these observations specifically for osteomyelitis, it should be noted that in other diseases this has already been documented. For example, management of patients infected with *S*. *aureus* bacteremia by an infectious disease specialist was found to reduce the mortality rate by 56% at 28 days post-therapy [23]. In addition, Rieg et al. [24] found that assessment of patients by the infectious disease department was a protective factor of mortality in a cohort of 521 patients with *S*. *aureus* infection (OR = 0.6 95% CI 0.4 to 1.0). This same impact was documented by Takakura et al. for Candidemia [25], and Schmitt et al. found a lower mortality (OR = 0.87 95% CI 0.83–0.91) and lower readmission (OR = 0.96 95% CI from 0.93 to 0.99) rates as well as less frequent admission to intensive care units [26].

One limitation of this study was that the sample size of the group treated by orthopedics only was lower than expected due to medical records that did not meet the inclusion criteria, which affected the accuracy of the results; this decreases the possibility of finding other variables that could be related. In addition, only 23 (17.82%) of patients completed a 12 month follow-up. Nevertheless, it should be noted that the follow-up of patients who relapsed was terminated immediately, and therefore the results of the first antibiotic cycle were not affected by new retreatment schemes in subsequent hospitalizations.

## Conclusions

The multidisciplinary management of osteomyelitis between the infectious disease specialist and the orthopedist increases cure rates while decreasing the likelihood of relapse. Our results encourage future studies in the same line of research with a probabilistic sampling in order to establish inferences in the general population and allow for evidence-based standardization of a treatment protocol.

## Supporting Information

S1 Database Supporting Information. (SAV)
